# Genome sequence of the yeast *Cyberlindnera sylvatica* UCD1060, isolated from soil in Ireland

**DOI:** 10.1128/mra.01162-24

**Published:** 2025-03-25

**Authors:** Padraic G. Heneghan, Adam P. Ryan, Sean A. Bergin, Margarita Caka, Aimee Coughlan, Julianna Decuseara, Michalina Gora, Daniil Hudov, Aoife McLoughlin, Sheila Osadolor, Isabel Saeed Maguire, Weronika Swistowska, Kenneth H. Wolfe, Geraldine Butler, Kevin P. Byrne

**Affiliations:** 1 School of Medicine, Conway Institute, University College Dublin 8797, Belfield, Ireland; 2 School of Biomolecular and Biomedical Science, Conway Institute, University College Dublin 8797, Belfield, Ireland; University of California Riverside, Riverside, California, USA

**Keywords:** yeasts, genome analysis

## Abstract

*Cyberlindnera sylvatica* is a member of the Cyberlindnera clade in the order Phaffomycetales. We present the genome sequence of *C. sylvatica*. The sequenced strain, UCD1060, was isolated from soil in Wicklow, Ireland. This genome is 13.5 Mb and was assembled into seven chromosome-sized contigs plus a mitochondrial genome contig.

## ANNOUNCEMENT

*Cyberlindnera sylvatica* is a recently characterized (1) species in the budding yeast order Phaffomycetales. It was first isolated in Hungary and Germany from forests. It has no known food or biotechnological applications. As it grows at 37°C, it could potentially be associated with mammals (1).

*C. sylvatica* UCD1060 was isolated from soil collected from the base of a large oak in the Devil’s Glen, County Wicklow, Ireland (GPS coordinates 53.0163025,–6.1524822) as part of an undergraduate research module (2).

Soil material was passaged twice at room temperature in 9 mL liquid yeast extract-peptone-dextrose (YPD) containing chloramphenicol (30 µg/mL) and ampicillin (100 µg/mL) and cultured on YPD agar plates. The species was identified by PCR and Sanger sequencing of the ribosomal DNA internal transcribed spacer (ITS) and D1/D2 regions (accession numbers PQ438548 and PQ438569), using primers ITS1 (TCCGTAGGTGAACCTGCGG) and ITS4 (TCCTCCGCTTATTGATATGC) (3), and for the D1/D2 region NL1 (GCATATCAATAAGCGGAGGAA) and NL4 (GGTCCGTGTTTCAAGACGG) (4). Sequence identity was 98.36% (540/549 bp) in the ITS and 99.96% (555/557 bp) in the D1/D2 region to the type strain of *C. sylvatica* (accession numbers MT305876 and MT316316; note MT305876 is misnamed in GenBank as “*Hyphopichia lachancei*” but comes from CBS 16335, the type strain of *C. sylvatica* [1]).

DNA was isolated by phenol/chloroform extraction from liquid YPD cultures grown at 20°C. Short-read library construction (Illumina DNA-Prep(M) Ref:20060060) and sequencing (UCD Conway Core Facility, Dublin, Ireland) used a NextSeq2000 and P1 flowcell, yielding 5.7 million read pairs (2 × 150 bp; 127× coverage). Adapters and low-quality reads were removed (Skewer version 0.2.2) (5). For long-read sequencing, after DNA extraction (Biosearch Technology Masterpure yeast DNA purification kit MPY80010), library preparation (Native Barcoding Kit SQK-NBD112-24), end repair (NEB-M6630), and ligation (NEB-E6056), we selected for DNA >3 kb with Long Fragment Buffer before Oxford Nanopore sequencing (MinION MK1C, flowcell FLO-MIN112 R10.4). Default fast basecalling was done on the instrument (MinKNOW version 23.07.12; default demultiplexing; barcode trimming; reads ≥500 bp kept; no quality cutoff; 74× coverage). NanoFilt (version 2.3.0) (6) retained 70,996 reads with quality ≥10 and length ≥1,000 bp (reads *N*_50_ = 21,004 bp), which were then assembled using Canu (version 2.2) (7), followed by five rounds of error correction using NextPolish (version 1.4.1) with the Illumina reads (8). Default parameters were used, except where otherwise noted.

The assembly consisted of seven nuclear contigs (total 13.5 Mb; *N*_50_ = 2,385,092 bp) and the mitochondrial genome (33,689 bp circular unit; accession number CAXWVZ010000008). Six nuclear contigs terminate with tandem repeats of (GGGTGTCT)_n_ at both ends, so they are inferred to be complete chromosomes with telomeres (9). The seventh nuclear contig (CAXWVZ010000007) has this telomeric repeat at one end and an rDNA array at the other end; this is the only rDNA locus in the genome.

Using BUSCO version 5.1.2 (10), genome completeness was estimated as 97.36% compared to the Ascomycota lineage data set. G + C content is 45.92%.

## Data Availability

This whole-genome shotgun project has been deposited as DDBJ/ENA/GenBank accession number CAXWVZ010000000 (BioProject no. PRJEB79408). The version described in this paper is version 1. The reads were deposited at ENA (accessions ERR13731774 and ERR13731842). The ITS sequence is PQ438548. The D1/D2 sequence is PQ438569. Isolate UCD1060 has been deposited in the CBS and PYCC culture collections as CBS 18652 and PYCC 10019.
